# Application of Digital Image Based on Machine Learning in Media Art Design

**DOI:** 10.1155/2021/8546987

**Published:** 2021-11-22

**Authors:** Ciguli Wu

**Affiliations:** Xi'an Jiaotong University City College, Xi'an, Shanxi 710068, China

## Abstract

In digital media art, expressive force is an important art form of media. This paper studies digital images that have the same effect when applied to media art. The research object is media art images, and the application effect of the proposed algorithm is related to the media art images. The development of digital image technology has brought revolutionary changes to traditional media art expression techniques. In this paper, a partial-pixel interpolation technique based on convolutional neural network is proposed. Supervised training of convolutional neural networks requires predetermining the input and target output of the network, namely, integer image and fractional image in this paper. To solve the problem that the subpixel sample cannot be obtained, this paper first analyzes the imaging principle of digital image and proposes a subpixel sample generation algorithm based on Gaussian low-pass filter and polyphase sampling. From the perspective of rate distortion optimization, the purpose of pixel motion compensation is to improve the accuracy of interframe prediction. Therefore, this paper defines pixel motion compensation as an interframe regression problem, that is, the mapping process of the reference image integral pixel sample to the current image sample to be encoded. In this paper, a generalized partial-pixel interpolation model is proposed for bidirectional prediction. The partial-pixel interpolation of bidirectional prediction is regarded as a binary regression model; that is, the integral pixel reference block in two directions is mapped to the current block to be coded. It further studies how to apply the trained digital images to media art design more flexibly and efficiently.

## 1. Introduction

With the development of communication technology, big data, and multimedia technology, multimedia applications have played an increasingly important role in people's lives. With the popularity of mobile terminals and the growth of video resolution, the amount of video data transmitted on the Internet is increasing rapidly, which brings unprecedented challenges to video coding. In recent years, artificial intelligence technology represented by machine learning has made great breakthroughs in image processing, computer vision, and natural language understanding. Deep neural network has strong nonlinear expression ability and can realize joint optimization by end-to-end training. How to combine machine learning technology with video coding and further improve the performance of digital images by machine learning is a valuable subject.

Existing video coding standards adopt interframe prediction technology based on motion compensation to remove temporal redundancy, thus reducing the coding rate of blocks to be encoded. Because of digital sampling, the actual motion of an object cannot be aligned with the sampling grid, so it is difficult to find an accurate matching block in the reference frame. In order to solve this problem, the concept of subpixel motion compensation is introduced in video coding, and the subpixel image is interpolated from the whole pixel image by interpolation filter, and the obtained subpixel image is used for motion compensation. This paper mainly studies how to use machine learning technology to learn more efficient interpolation filters, so as to improve the coding performance of subpixel motion compensation. Digital images are virtual beyond reality and can be used to produce stereovisual effects. In this design, the optimized subpixel interpolation model and motion compensation model are used to realize the feature expression ability of digital media art design; the integration of comprehensive information technology can effectively improve the visual expression ability of digital media art design system to match key information points. Traditional digital media art design system has poor visual performance ability. In order to solve this problem, this paper uses CNN model to reconstruct three-dimensional image features.

After several years of development, digital image not only has expanded under the impetus of scientific research, but also has been combined with computational media art to have layering, which extends image beyond the concept of visual surface. Contemporary imaging contributes to the field of digital media art (DMA), and artists use biometric data, artificial intelligence, and other tools to explore the intermediary elements contained in postimages [1]. The application characteristics and advantages of computer vision art are quite obvious, and it is mainly used in image information processing, animation information processing, game picture processing, and so on [2]. Combined with the current cultural connotation transmission and intelligent development trend, we need to study and exert its value and improve the development speed of digital media art. The change of media communication mode has played a great role in promoting [3]. It can be considered that digital media art represented by computers has had a far-reaching impact on today's animation design. First of all, digital media has inherited the characteristics of various arts and has formed its own artistic characteristics. On this basis, the status of animation design and its impact are analyzed, and then the purpose of its research is that computer digital media can play a positive role in animation design. Holographic projection technology is the most representative and popular application technology in the field of digital media art in the digital age. We first analyze the principle and application of holographic projection technology, then dig deep into its advantages in image, and finally propose introducing it into digital media art applications such as digital art museums and digital art exhibitions [4]. After verification, holographic projection technology can be used in art exhibitions and 3D image recording, which is not only recognized by art-loving audiences, but also provides high-quality experience for audiences who cannot watch on the spot. In the digital age and the background of big data, although digital media has developed rapidly driven by big data, its development has been hindered due to technical limitations, such as shortage, backwardness, and lack of support, especially the lack of cross-application [5]. It is beneficial to study the cross-teaching under the background of big data. Through the current situation of digital media art teaching, research was launched to seek solutions and put forward scientific basis for the development of recommendations and guidance. Today, with the gradual prevention of the application of digital media art, we continue to study the preliminary application of deep learning method in intelligent electronic music system [6] and propose a DDPG algorithm based on multitask learning (M-DDPG) based on deep deterministic strategy gradient (DDPG). It proposes a hierarchical learning-based DDPG algorithm (H-DDPG) for the image hierarchy problem. After evaluation, M-DDPG algorithm is better than H-DDPG algorithm, and the test results are more accurate. However, H-DDPG algorithm is ideal for complex tasks. They have their own performances in image processing of intelligent systems and have great potential. With the emergence of intelligent machine learning, people's requirements for robots are no longer simple movement and language, but a smarter and simpler form of human-computer interaction. This also brings a new driving force for the development of image vision, thereby strengthening the importance of machine learning in today's science and technology. Machine learning technology is the mainstream algorithm in the field of science and technology, especially in the field of computer vision images. Compared with traditional image recognition methods, machine learning technology can achieve higher accuracy. In addition, compared with traditional methods, machine learning method can provide stronger stability. At the same time, machine learning technology can also correlate visual and semantic information, thus constructing semantic database of visual motion environment, providing intelligent computing for cognition and task reasoning, thus providing higher intelligence for visual image mechanism. The second part of the article introduces the related knowledge of machine learning and studies the theoretical basis of the model in the media; the third part considers the specific goal of the application of machine learning method for the form of artistic expression of digital media; the fourth part introduces the subpixel interpolation technology based on convolution neural network. The fifth part compares the methods used in experiments and compares the algorithm effects of different objects by grouping.

## 2. Machine Learning

Machine learning algorithm is an algorithm that automatically learns useful information from data. A formal definition of “learning” is given from the point of view of computer program [7]: if *P* is the performance measure criterion of task *T*, and if a computer program can improve the performance criterion *P* of task *T* by using experience *E*, it is said that for task *T* and performance measure *P* the program has learned from experience *E*.

Generally, there are two aspects to consider in the performance evaluation of machine learning algorithms [8]. On the one hand, it is necessary to design performance evaluation criteria for tasks themselves. The performance evaluation index of the task itself needs to be designed according to the specific task.

### 2.1. Feedforward Neural Network

Feedforward neural network is one of the simplest machine learning models [9], and it is also the basis of other machine learning models. The purpose of feedforward neural network is to learn a mapping *y* = *f* (*x*; *θ*) by adjusting the parameter *θ* of the network for input (*x*; theta) to approximate the real function *f*^*∗*^ from input to output.

#### 2.1.1. Neuron Model

**Figure 1:**
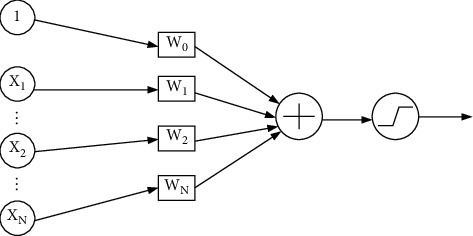
Neuron model.

The feedforward neural network is inspired by biological neurons. The simplest feedforward neural network is called single layer perceptron, also called neuron model, as shown in Figure 1. The relationship between output and input of neuron model can be expressed as follows.

(1) $$\begin{array}{c} y=\sigma \left(\sum_{i=1}^{N}w_{i}\times x_{i}+w_{0}\right). \end{array}$$

The neuron model consists of two parts [10]: weighted summation and activation function. The activation function *σ* (∙) makes neurons have nonlinear ability, thus improving the expression ability of neurons. Neuron model can only deal with linearly separable data. For complex learning problems, it is necessary to build more complex multilayer neural networks.

#### 2.1.2. Multilayer Perceptron

**Figure 2:**
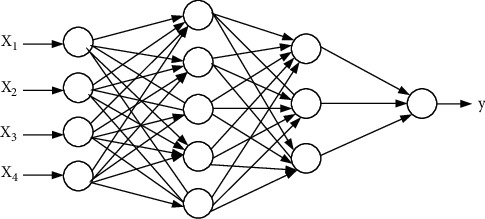
Schematic diagram of feedforward neural network model.

Feedforward neural network, also known as multilayer perceptron, adopts unidirectional multilayer structure, in which each layer contains several neurons. Neurons in each layer receive signals from the previous layer and send the processed signals to neurons in the next layer [11, 12].  Figure 2 shows a schematic diagram of feedforward neural network. Feedforward neural network has an input layer, an output layer, and several hidden layers in the middle. In Figure 2, the input to the network is a vector of *n*_0_ dimension

(2) $$\begin{array}{c} \overset{⟶}{x}={\left[x_{1},x_{2},\ldots ,x_{n_{0}}\right]}^{T}. \end{array}$$

The final output of the network is *y*. The relationship between input and output of layer *q* is defined as

(3) $$\begin{array}{c} x_{i}^{q}=\sigma \left(\sum_{j=0}^{n_{q}}w_{ij}^{q}\times x_{ij}^{q-1}\right). \end{array}$$

### 2.2. Convolution Neural Network

Convolution neural network introduces three important characteristics [13]: sparse connectivity, parameter sharing, and equivariant representation [14].

**Figure 3:**
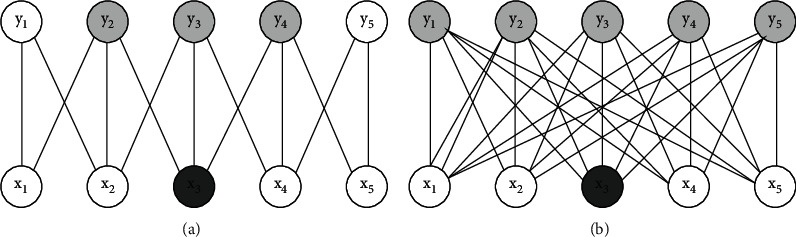
Schematic diagram of sparse connection (from bottom to top). (a) Sparse connectivity. (b) Full connection.

**Figure 4:**
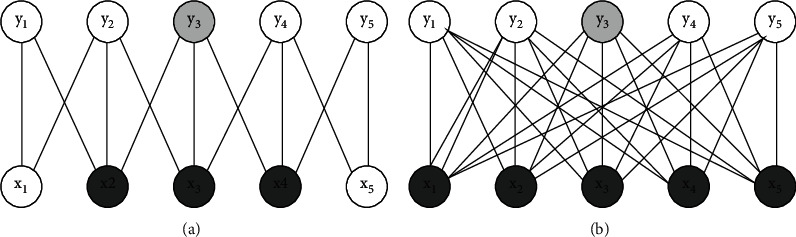
Schematic diagram of sparse connection (from bottom to top). (a) Sparse connectivity. (b) Full connection.

Feedforward neural networks use matrix multiplication to express the connection between inputs and outputs, in which each output is related to all inputs. Convolution neural network uses a sparse connection mode, which makes each output unit only related to a local area of input by reducing the size of operation core [15]. For example, when processing an image, the input image may have thousands of pixels, but we may only need to use the kernel to detect some small meaningful features (such as edges). Therefore, through sparse connection, convolution neural network only needs to store small parameters, which reduces the storage overhead and computational complexity. Figures 3 and 4 show the connection schematic diagram of convolution neural network and feedforward neural network. The left diagram of Figure 3 uses sparse connection, output *y* is obtained by convolution operation with kernel size 3, and only three output cells are affected by input cell *x*_3_. The right figure of Figure 3 does not use sparse connection, and each output unit is affected by input unit *x*_3_. As can be seen from the left diagram of Figure 4, the output unit *y*_3_ is affected by three units in the input, which are called the receptive field of *y*_3_. In the right figure of Figure 4, *Y* is obtained by matrix multiplication, and each input cell will have an impact on the output *y*_3_.

**Figure 5:**
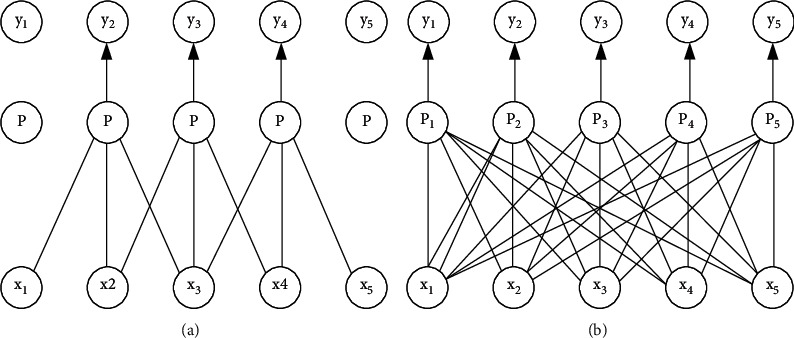
Schematic diagram of parameter sharing of convolution neural network. (a) Parameter sharing. (b) No parameter sharing.

Parameter sharing in convolution neural network means that multiple functions in the model use the same parameters. In a feedforward neural network, each output unit has its own set of parameters, and the output unit is obtained by convolving this set of parameters with the input. In convolution neural networks, elements of convolution kernel are used at every input position.  Figure 5 shows the comparison between parameter sharing and nonparameter sharing. It can be seen that all output units *y*_*i*_ in the method using parameter sharing share a set of parameters *p*; in the scheme without parameter sharing each output unit *y*_*i*_ uses a separate set of parameters *p*_*i*_.

In convolution neural network, the parameter sharing property makes the convolution layer have translation and other degeneration. A function has equivariance, which means that if the input changes, the output will change accordingly. For the functions *f* and *g*, if *f*(*g*(*x*))=*g*(*f*(*x*)), then *f* is said to be equivalent to *g*. For example, if the object in the input image is translated, the expression in the output will be translated in the same way.

After extracting local features by convolution calculation, convolution neural network also needs to map convolution results by activation function to get output. The use of activation function introduces nonlinear mapping ability to the convolution neural network model, thus improving the expression ability of the model. Commonly used activation functions are Sigmoid function, hyperbolic tangent function, ReLU, and so on. Sigmoid function and hyperbolic tangent function:

(4) $$\begin{array}{c} \text{sigmoid}\left(x\right)=\frac{1}{1+e^{-x}},\\ \tanh\left(x\right)=\frac{e^{x}-e^{-x}}{e^{x}+e^{-x}}. \end{array}$$

ReLU factor activation function expression:

(5) $$\begin{array}{c} \text{ReLU}\left(x\right)=\max\left(0,x\right). \end{array}$$

However, when the input of ReLU activation function is negative, the gradient is still zero. To solve this problem, ReLU is improved, and its expression is

(6) $$\begin{array}{c} \text{LReLU}\left(x\right)\left\{\begin{array}{cc} x, & \text{if }x>0,\\ ax, & \text{if }x>0. \end{array}\right. \end{array}$$

## 3. Media Art Design

The world is about to enter the peak of digital information technology development. The 19th National Congress also put forward the core content of the following: “innovation is the primary productive force”. Scientific and technological innovation will become a great driving force for urban construction. Digital media art, as a field of combining science with art, will guide the design concept of urban landscape facilities from the initial concept of combining function with form to the design concept of service, interaction, experience, intelligence, and diversification. This change of concept makes the innovation of urban landscape design pay more attention to the system innovation of intelligent service, diversified functions, and intensive management on the basis of form and function innovation. Also, it enhances the interactive experience between landscape facilities and citizens and emphasizes green design and low-carbon design. The development of digital media art promotes the innovation and perfection of urban landscape design and realizes the green ecology, intelligent management, and systematic information service of urban landscape design. The innovation of urban landscape facilities will continuously meet the new needs of public travel, work, life, information, and entertainment, continuously optimize urban functions, update urban image, change urban life, and improve urban management level. In digital media art, “digital” reflects that digital media art is based on science and technology, “media” emphasizes that it belongs to multimedia industry, and “art” indicates that digital media art is mainly used for art design and digital multimedia art design. Digital media art not only involves graphic design, art design, landscape design, interactive design, and other art design fields, but also involves digital information technology, computer language, computer programming, digital information transmission technology, and other scientific and technological fields. The application form is visual art or design works created by means of digital media, digital technology, and other fields of science and technology. It is the most innovative subject in the field of art design at present.

### 3.1. Main Features of Digital Media Art

tFirst of all, compared with traditional media art, the most essential feature of digital media art is that it is more shared, open, and compatible, and its artistic expression forms are more diverse, with stronger artistic appeal, visual impact, and interactive experience. Secondly, the combination of digital information technology and other scientific and technological means in the process of design and creation and the form of artistic expression is also the key point that distinguishes digital media art from other art forms. Furthermore, digital media art is not a single media art creation, but a combination of various media arts, so digital media is also called “multimedia.”

### 3.2. Classification of Digital Media Art

#### 3.2.1. Static Digital Art

Static digital art is an early art form. It mainly refers to static pictures in digital forms such as single frame pictures, printed products, and digital pictures and includes digital works such as computer painting creation and computer postimage processing. No matter what form of art pictures is static, this is the earliest digital art form.

#### 3.2.2. Dynamic Digital Art

Dynamic digital art includes digital video and two-dimensional and three-dimensional animation. With the help of FLASH, PHOTOSHOP, and other drawing software programs, it integrates various art forms of sound, light, color, and beauty and successfully attracts the public's attention in various public places, with excellent creative performance effect. The artistic image of dynamic digital art is much higher than that of traditional artistic creation, and the image is more vivid and lifelike. It is being used more and more in digital image design and has become the most popular digital art form today.

#### 3.2.3. Interactive Experience Art

**Figure 6:**
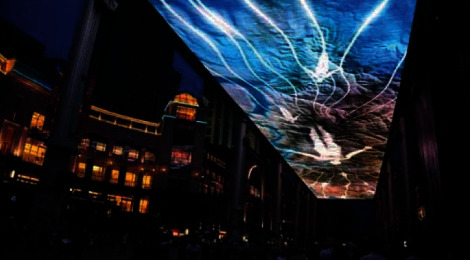
Beijing electronic dream sky curtain.

Interactive art mainly refers to word media installation art, which includes sensors, infrared recognition, and information transmission technologies. Interactive art works are mainly displayed in urban public spaces, science and art venues, or museums. Interactive art appears in the form of intelligent interaction combining various transmission modes and digital media, which makes the audience feel the design concept that the works of art want to convey more truly. This interactive art work can recognize and capture people's vision, smell, touch, taste, hearing, and other feelings through computers. It can also capture language, expression, facial movements, and body movements and complete real-time feedback, so as to realize the interaction between audience and works. Interactive experience art is the most popular artistic expression in urban public space at present, as shown in Figure 6.

### 3.3. Computer-Generated Holography

**Figure 7:**
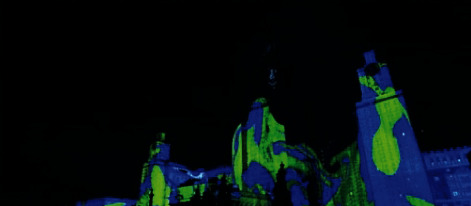
2016 Moscow 3D mapping wall show.

Computer-generated holography is an organic combination of optical holography and computer technology. The 2D complex amplitude distribution of a three-dimensional object on the holographic plane is simulated by computer and encoded into a computer-generated hologram, which is finally loaded into a Spatial Light Modulator (SLM), and the real three-dimensional image of the object is presented in space by the reconstructed light. Computer-generated holography 3D display technology can record and reproduce real 3D objects and is also applicable to virtual 3D objects. By computer simulation instead of the construction of optical system, the requirements of optical equipment are reduced and the operation is simplified, which has high flexibility and repeatability. Therefore, computer-generated holography 3D display technology is the frontier direction of digital image display, media art design, and large data storage technology. In recent years, it has been widely valued in theoretical and applied research, as shown in Figure 7.

## 4. Subpixel Interpolation Technology Based on Convolution Neural Network

In modern media art design, digital images, as an important information carrier, play a more and more critical role in the production and life of human society. The research on software and hardware technology involved in digital image acquisition, storage, transmission, and processing has been widely concerned. In this chapter, fractional pixel-based motion compensation technology is widely used in video coding standards, which can significantly improve the rate distortion performance of interframe prediction. The core of fractional pixel motion compensation is to design an efficient fractional pixel interpolation filter. The function of interpolation filter is to interpolate integer pixel images to generate fractional pixel images. Generally speaking, a good interpolation filter can play two roles. Firstly, the spectral aliasing caused by digital sampling can be removed, so that the samples at fractional positions can be reconstructed as much as possible; secondly, the interpolation filter needs to be able to suppress the coding noise to a certain extent in order to solve the problem of coding noise in the reference frame of lossy video coding. Because most natural images are nonstationary signals and the coding noise is often nonlinear, the traditional linear interpolation filter based on fixed taps is difficult to meet the above two conditions [16]. In this chapter, in order to improve the expression ability of interpolation filter and further improve the rate distortion performance of coding, the author studies the fractional pixel interpolation technology based on convolution neural network. Aiming at the problem that fractional pixel samples cannot be obtained due to digital sampling, the author proposes a fractional pixel sample generation algorithm based on Gaussian low-pass filtering by analyzing the principle of digital image formation. Based on the generated fractional pixel samples, the author further studies the generation algorithm of interpolation filter training data. In addition, the author proposes using convolution neural network to train efficient interpolation filters and designs the corresponding training algorithm.

### 4.1. Generation Method of Subpixel Samples Based on Gaussian Low-Pass Filtering

**Figure 8:**
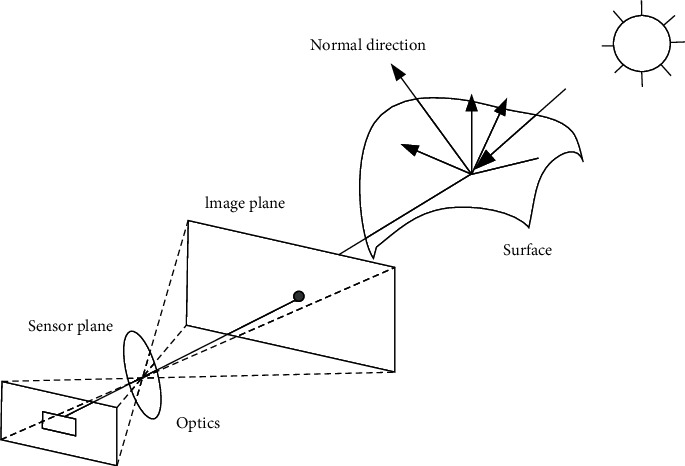
A simplified optical image forming process is shown. Light is emitted from a light source and then reflected through the surface of an object. Part of the light goes directly into the camera.

The first step of training interpolation filter based on convolution neural network is to obtain the training data needed for supervised training, so this paper first studies how to obtain fractional pixel samples and corresponding integer pixel samples. Based on the principle of image formation, this paper reviews and analyzes the image formation model [17, 18]. Images are composed of discrete luminance values, which are related to light in physical environment [19], surface characteristics of objects, geometric characteristics of objects, and optical characteristics of cameras.  Figure 8 is a schematic diagram of a simplified optical image forming process. The light is emitted from the light source and irradiates the surface of the object. After being reflected by the surface of the object, a part of the light directly enters the camera, and the photoelectric conversion is realized on the sensor.

**Figure 9:**
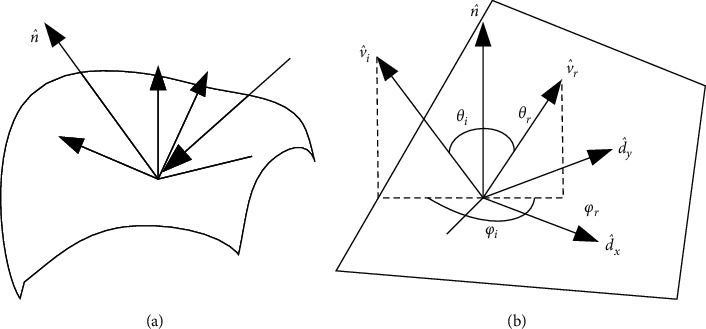
(a) Light is scattered by the surface of an object. (b) The bidirectional reflection distribution function *f*(*θ*_*i*_, *φ*_*i*_, *θ*_*r*_, *φ*_*r*_) is a four-parameter model of the angle between the incident direction ${\hat{v}}_{i}$ and the reflection direction ${\hat{v}}_{r}$ and the local plane coordinate $\left({\hat{d}}_{x},{\hat{d}}_{y},\hat{n}\right)$.

Light sources can generally be divided into point light sources and area light sources. A point light source emits from a certain position in space (or from an infinite distance, such as the sun). In addition to spatial position, point light sources have two attributes: brightness and color spectrum *L*(*λ*) (i.e., wavelength distribution). Because the light diffuses along the sphere, the brightness of the light source can be regarded as decaying according to the square of the distance between the light source and the object. Surface light source is more complex than point light source. A commonly used method to approximate incident illumination is called reflection mapping model, which maps incident light direction $\hat{v}$ to color value $L\left(\hat{v},\lambda \right)$. When light shines on the surface of an object, it will be scattered and reflected, as shown in Figure 9(a). There are many models to describe the scattering and reflection of light, among which the most basic model is the bidirectional reflection distribution function. As shown in Figure 9(b), the bidirectional reflection distribution function describes how much of the light incident in the direction ${\hat{v}}_{i}$ is reflected in the direction ${\hat{v}}_{r}$. The bidirectional reflection distribution function can be written as a function of the angle of the incident direction and the reflection direction relative to the tangent coordinates of the illumination point, that is, *f*_*r*_(*θ*_*i*_, *φ*_*i*_, *θ*_*r*_, *φ*_*r*_, *λ*).

The surfaces of many objects are isotropic; that is, the probability of light propagating in all directions is the same. For isotropic materials, the bidirectional reflection distribution function can be simplified as $f_{r}\left({\hat{v}}_{i},{\hat{v}}_{r},\hat{n},\lambda \right)$. In order to calculate all the illumination reflected along the ${\hat{v}}_{r}$ direction at a certain point on the surface of the object under a given illumination condition, the incident light $L_{i}\left({\hat{v}}_{i},\lambda \right)$ can be integrated (i.e., convoluted) with the result of the bidirectional reflection distribution function, and the following results can be obtained:

(7) $$\begin{array}{c} L_{r}\left({\hat{v}}_{i};\lambda \right)=\int L_{i}\left({\hat{v}}_{i};\lambda \right)f_{r}\left({\hat{v}}_{i},{\hat{v}}_{r},\hat{n};\lambda \right)\cos^{+}\theta_{i}d{\hat{v}}_{i}, \end{array}$$

where cos^+^*θ*_*i*_ = max (0, cos*θ*_*i*_). If the light rays are discrete, that is, there are only finite point light sources, then the integration can be replaced by summation:

(8) $$\begin{array}{c} L_{r}\left({\hat{v}}_{i};\lambda \right)=\sum_{i}L_{i}\left(\lambda \right)f_{r}\left({\hat{v}}_{i},{\hat{v}}_{r},\hat{n};\lambda \right)\cos^{+}\theta_{i}. \end{array}$$

The reflected and refracted illumination enters the camera, reaches the sensor through the lens, and finally forms the digital image that we see through a series of operations such as exposure, sampling, and denoising. From formulas (7) and (8), it can be seen that the image forming process can be simplified as the convolution of the original analog signal and the bidirectional reflection distribution function. The convolution operation in spatial domain corresponds to filtering operation in frequency domain. In other words, the process of digital image generation can be simplified into two processes: filtering and sampling. Although the actual digital image formation process is much more complex than that described in the above model, the above analysis still inspires us to design fractional pixel generation algorithms.

### 4.2. Network Structure and Training Algorithm Adopted

SRCNN consists of three convolution layers, and the output of each layer is the result of convolution and nonlinear transformation of the previous layer [20]. The nonlinear transformation used here is a linear rectifier unit. The specific operation is described as follows:

(9) $$\begin{array}{c} F_{i}=\max\left(0,W_{i}*F_{i-1}+B_{i}\right), \end{array}$$

where *W*_*i*_ and *B*_*i*_ are the convolution kernel and offset of layer *i*, respectively, and ^*∗*^ represents convolution operation.

### 4.3. Subpixel Motion Compensation Technology Based on Interframe Regression Model

In digital media art design [21], it is necessary to collect sufficient projection images and accurately extract spectrum information on paraboloid of revolution by using multiview projection images to extract spectrum sampling and generate computer-generated holograms. At present, multiview projection images of three-dimensional objects are mainly obtained by interframe regression model, which simplifies the shooting process and reduces the number of projection images. The core of subpixel motion compensation technology [22] is to design efficient interpolation filter. The motion compensation of subpixel precision is modeled theoretically, and it is pointed out that the performance improvement of subpixel interpolation filter comes from two aspects: precision effect and filtering effect. Accuracy effect means that interpolation filter makes motion compensation use more accurate motion vector; the filtering effect is mainly to remove the coding noise of the reference frame. Therefore, the subpixel motion compensated interpolation filter can be decomposed into two filtering operations, one is a pure value-pumping operation, and the other is to remove coding noise. The whole of these two filtering operations can be Wiener filtering. In other words, the function of the subpixel motion compensation is to make the subpixel image generated by interpolation as similar as possible to the current image to be encoded.

In this chapter, we propose a novel definition of subpixel motion compensation. As we will see, this new definition allows us to avoid the problem that subpixel samples cannot be obtained [23]. In order to be consistent with block-based video coding schemes (such as HEVC), we consider how to predict the current block to be encoded from a given reference image. The block currently to be encoded is represented as *B*_cur_, and the reference image is represented as *P*_ref_. Since we are considering the subpixel motion compensation problem, we can assume that the optimal motion vector has been determined and the optimal motion vector is fractional motion vector. Now, we need to solve the optimal subpixel reference block generation function, which can predict the current block to be encoded as accurately as possible.

(10) $$\begin{array}{c} F^{*}\equiv \arg\underset{F\in \Omega }{\min}D\left\{F\left(P_{\text{ref}},mv\right)\right.,\left.B_{\text{cur}}\right\}. \end{array}$$

Because of the variety of motion vectors in natural video, it is difficult to mathematically describe the generating function F (MV). Therefore, we transform the above problem into a looser problem; that is, we assume that the subpixel reference generation function is invariant to the integer pixel translation. We can decompose the motion vector into an integer part and a fractional part; that is, *mv* = *mv*_*i*_ + *mv*_*f*_, *x*_*i*_, *y*_*i*_ ∈ *Z*, *mvf* = (*x*_*f*_, *y*_*f*_), 0 ≤ *x*_*f*_, *y*_*f*_ ＜ 1, so the problem becomes

(11) $$\begin{array}{c} F^{*}\equiv \arg\underset{F\in \Omega }{\min}D\left\{F\left(P_{\text{ref}}⊕mvi,mvf\right),B_{\text{cur}}\right\}, \end{array}$$

where *P*_ref_ ⊕ *mvi* represents moving the reference image according to *mvi* (equivalent to changing the original coordinate position).

In practical video coding schemes, the fractional accuracy of motion vectors is limited, so we can further simplify the problem by decomposing the above generation function into several simple generation functions, each of which is only used to generate pixel values at a certain fractional position.

(12) $$\begin{array}{c} f_{\text{mvf}}^{*}\equiv \arg\underset{f_{\text{mvf}}\in \Omega_{\text{mvf}}}{\min}D\left\{f_{\text{mvf}}\left(P_{\text{ref}}⊕mvi\right),B_{\text{cur}}\right\}, \end{array}$$

where *f*_mvf_(·) is the generating function corresponding to a fractional position and Ω_*mvf*_ is the corresponding function space. For example, if we only consider motion compensation with half-pixel accuracy, then mvf ∈ {(0, 1/2), (1/2, 0), (1/2, 1/2)}, corresponding to three kinds of generating functions. If we consider motion compensation with quarter accuracy, then there are 15 generation functions corresponding to different fractional positions.

In the field of machine learning, the standard regression problem is generally shown in the following formula:

(13) $$\begin{array}{c} f^{*}\equiv \arg\underset{f\in \Delta }{\min}L\left(f\left(X\right),Y\right), \end{array}$$

where *f* is the return function, the input variable is *X*, the target variable is *Y* which represents the corresponding function space, and *L* (∙, ∙) is the loss function. Comparing formula (12) with formula (13), we can see that the fractional pixel motion compensation problem of unidirectional prediction proposed in this chapter is mathematically equivalent to regression problem, in which the input variable *X* corresponds to *P*_ref_ ⊕ *mvi* and the target variable corresponds to *B*_cur_. Therefore, the optimal fractional pixel sample generation function can be solved by the general regression problem. We solve the above problems from the perspective of machine learning. Firstly, we collect many training data pairs ∑_*i*_*L*(*f*(*X*_*i*_), *Y*_*i*_), then determine the function space Δ of regression function in advance, and solve the optimal regression function *f*^*∗*^ by minimizing the total loss function.

In this chapter, we propose a method based on CNN model training to solve the above regression problem. The function space is determined by the structure of the adopted CNN model, and the regression function is expressed by a set of network model parameters, so there are

(14) $$\begin{array}{c} \theta^{*}\equiv \arg\underset{\theta }{\min}\sum_{i}D\left\{\left(X_{i}|\theta \right)\right.{,}_{i}\left.Y\right\}, \end{array}$$

where *h* represents CNN model and *θ* represents CNN model parameters. Formula (14) can be solved by a training algorithm of a standard CNN model, such as a statistical gradient descent algorithm.

In this chapter, from the perspective of interframe image regression, we think that bidirectional motion compensation is different from unidirectional motion compensation. Accordingly, in this chapter we extend the definition of unidirectional subpixel motion compensation to bidirectional motion compensation prediction as shown in

(15) $$\begin{array}{c} g_{\text{mvf}1,\text{mvf}2}^{*}=\arg\underset{g\in \psi }{\min}\left\{g_{\text{mvf}1,\text{mvf}2}\right.\left(P_{\text{ref}1}⊕mvi_{1},P_{\text{ref}2}⊕mvi_{2}\right),\left.B_{\text{cur}}\right\}, \end{array}$$

where *P*_ref1_ and *P*_ref2_ denote two reference images for bidirectional prediction, *mvi*_1_ and *mvi*_2_ denote integer portions of motion vectors corresponding to two reference frames, mvf1 and mvf2 denote fractional portions of motion vectors in both directions, *g* denotes fractional pixel reference generation functions for bidirectional motion compensation, and *ψ* denotes corresponding function spaces.

## 5. Experiment

### 5.1. Algorithm Performance Based on HEVC Reference Software

The machine learning framework Caffe is used to train the convolution neural network, and the NVIDIA Tesla K40C GPU is used as the computing platform. The training set includes 400 natural images, all of which generate integer and fractional pixel samples through the above method, finally get 400 subimages as integer pixel training data, and get 400 images corresponding to each subpixel position as training label data. The trained interpolation filter is integrated into HEVC reference software HM (16.7 version) to directly replace the half-pixel DCTIF interpolation filter. The corresponding model is selected according to the slice level QP of each reference frame; specifically, the model corresponding to the nearest QP of {QP22, QP27, QP32, QP37} will be used as the interpolation filter model of the reference frame. The experiment tested the low delay P (LDP) configuration according to the HEVC general test condition and adopted BD-rate as the measure of rate distortion performance.

PSNR is call for peak signal to noise ratio. The larger the PSNR is between two images, the more similar it is. The general reference is 30 dB, and the degradation of images below 30 dB is obvious.

(16) $$\begin{array}{c} \text{PSNR}=10\;\log_{10}\left(\frac{{\text{MAX}}^{2}}{\text{MSE}}\right). \end{array}$$

MAX is the maximum value of the image color and is 255 for 8 bits.

(17) $$\begin{array}{c} \text{MSE}=\frac{1}{mn}\sum_{i=1}^{n}\overset{m}{\underset{j=1}{\sum }}{\left\|K\left(i,j\right)-I\left(i,j\right)\right\|}^{2}. \end{array}$$

Structural similarity index (SSIM) algorithm takes into account the visual characteristics of human eyes in design, which is more in line with human visual perception than traditional methods. SSIM can be calculated based on different windows, assuming that the sizes of windows *x* and *y* are *N∗N*:

(18) $$\begin{array}{c} \text{SSIM}\left(x,y\right)=\frac{\left(2\mu_{x}\mu_{y}+c_{1}\right)\left(2\sigma_{xy}+c_{2}\right)}{\left(\mu_{x}^{2}+\mu_{y}^{2}+c_{1}\right)\left(\sigma_{x}^{2}+\sigma_{y}^{2}+c_{2}\right)}. \end{array}$$

BD-rate calculates the average value of the difference between the two RD curves corresponding to the above two algorithms. It is necessary to fit the curve of several (usually four) points tested, then make the difference, and finally take the average.

The BD-rate performance results of the proposed scheme compared with HEVC are shown in Table 1. It can be seen from the table that the CNNIF proposed in this paper has an average improvement of 0.9% in luminance component compared with the baseline results of HEVC. For some test sequences, such as BQTerrace [24], the performance improvement is 3.2%. It should be noted that because the motion vector accuracy of chrominance in HEVC is higher, in order to consider the complexity, only the half-pixel interpolation filter of luminance component is trained and replaced in this experiment, so the performance of chrominance component is not obvious. The results of this experiment verify the feasibility of applying convolution neural network to fractional pixel interpolation.

### 5.2. Analysis and Comparison with Superresolution Method

This paper uses convolution neural network to train interpolation filter, which is inspired by the application of machine learning in image superresolution. From the perspective of image resolution, both image superresolution and fractional pixel interpolation are to recover high-resolution images from low-resolution images, and there is no clear learning goal for these two problems. However, there are still two obvious differences between image superresolution and image fractional pixel interpolation. Firstly, the target of image superresolution is mainly from low-resolution high-resolution image, and fractional pixel interpolation of image not only restores high-resolution image, but also ensures that the pixels at the phase zero of high-resolution correspond to the pixels of the input image. Secondly, the input image of image superresolution problem is usually noise-free, but the input image of image fractional pixel interpolation is noisy. Therefore, it can be seen from the above analysis that the fractional pixel interpolation of images requires higher requirements and has more constraints than the superresolution problem of images, as shown in Table 2.

When SRCNN is used for image superresolution, it is necessary to use bicubic interpolation to upsample the low-resolution image first and then take the bicubic upsampled image as the input of the network to get the final high-resolution image. When SRCNN is used for fractional pixel interpolation, the input of network is integer pixel image (low resolution), and the input of network is fractional pixel image at a certain position (low resolution). It is true that by designing a network with appropriate upsampling mode all fractional pixel images can be directly recovered from the input integer pixel images. In this experiment, CNNIF and image superresolution method proposed in this subject are compared. First, the reference frame is twice upsampled using a pretrained SRCNN model, and then the upsampled image is polyphase sampled, and phase *L*, phase 2, and phase 3 are, respectively, used as half-pixel interpolated images at three positions. The image superresolution method is integrated into HM16.7, and the BD-rate performance results of Class C and Class D test sequences are given in Table 2. It can be seen that the half-pixel interpolation method based on superresolution will bring very obvious performance loss, and the performance loss of luminance component for BQSquare sequence reaches 8.2%. This experiment shows that although image interpolation and image superresolution are similar, the two methods are essentially different, so image superresolution cannot be directly used in subpixel interpolation of video coding.

### 5.3. Filter Type Merging Mode

**Figure 10:**
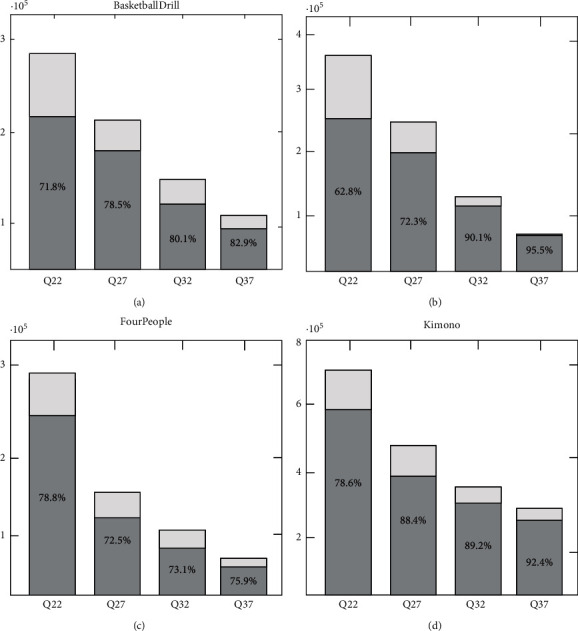
The number of coding units encoded using the Merge 2N × 2N mode and the proportion of correctly combined coding units.

HEVC video coding standard introduces a new motion information combining mode, which can effectively improve the coding efficiency of interframe prediction. In the combined mode, the current PU can inherit the motion information of adjacent blocks in spatial domain or temporal domain, which can effectively save the number of bits needed to encode this motion information compared with the general interprediction. Generally speaking, the smaller the current PU size is, the more likely it is to have the same motion information as the neighboring blocks. Similar to motion information, smaller blocks also tend to have the same subpixel reference filtering type as adjacent blocks. Firstly, we analyze and verify the above conjecture experimentally. Considering Merge 2N x 2N, we calculate the ratio of correct merging, where correct merging means that the merged subpixel reference filter type is the same as the filter type obtained by rate distortion optimization. The experimental results are shown in Figure 10. From the experimental results, we can see that the correct combination ratio can reach more than 70%. Therefore, when the encoding unit is encoded by Merge 2N × 2 N model, the corresponding subpixel reference filter type does not need to be obtained by rate distortion optimization but is directly inherited from adjacent blocks and does not need to be marked in the encoded bit stream.

### 5.4. Performance Verification of FRCNN Model with Bidirectional Prediction

**Figure 11:**
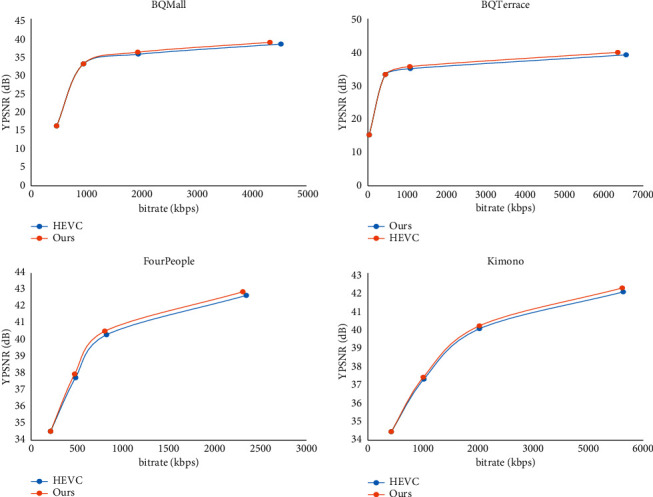
Comparison of rate distortion curves between the proposed FRCNN model and HEVC.

A notable feature of the subpixel motion compensation algorithm proposed in this paper is that two sets of FRCNN models are used for bidirectional motion compensation. We use experiments to verify the performance of using two sets of models in Figure 11.

For coding performance comparison, here we use only the FRCNN-U model for the bidirectional motion compensation mode; i.e., the FRCNN-U model is used for both reference image lists 0 and 1, as shown in Tables 3 and 4.

The encoded BD-rate performance results are shown in Table 3. It can be seen that, under LDB test conditions, using FRCNN-U and FRCNN-B at the same time can achieve 2.7% BD-rate performance improvement; only using the FRCNN-U model can only achieve a 2.0% improvement in BD-rate performance. For sequences with lower resolution, this performance difference is more obvious. The experimental results show that the bidirectional predictive FRCNN model proposed in this paper can effectively improve the rate distortion performance of bidirectional motion compensation.

In order to make the experiment illustrative, numerical comparison is used to compare the operation time of traditional media art design with that of FRCNN model based on bidirectional prediction in this paper, as shown in Table 5.

It can be seen from the test results that the bidirectional prediction FRCNN model takes less time; the target detection task is correct and can be completed stably, which meets the requirements of digital media art design.

The experimental results show that the bidirectional prediction FRCNN model can effectively improve the rate distortion performance of bidirectional motion compensation and take less time. In digital media art design, it is necessary to collect sufficient projection images and accurately extract spectrum information on paraboloid of revolution by using multiview projection images to extract spectrum sampling and generate computer-generated holograms. At present, multiview projection images of three-dimensional objects are mainly obtained by interframe regression model, which simplifies the shooting process and reduces the number of projection images. The core of subpixel motion compensation technology is to design efficient interpolation filter. The bidirectional predictive FRCNN model can improve the rate distortion performance of bidirectional motion compensation effectively, so that the precision effect and filtering effect of the subpixel interpolation filter can be improved.

## 6. Conclusion

Various input objects are not only the starting point of digital media art creation platform, but also an important part of the system. In the application of digital media art design, the input objects mainly include input devices and user preset data. Image and video are the main input elements in the system. Combined with the development trend of digital media art design, machine learning and intelligent optimization algorithm are introduced, and a new subpixel sample generation method based on Gaussian low-pass filtering and subpixel interpolation technology based on convolution neural network are studied. The experimental results show that the interpolation filter algorithm based on convolution neural network and the interframe regression model based on subpixel motion compensation proposed in this paper can meet the practical application requirements in media art design.

## Figures and Tables

**Table 1:** BD-rate performance results of this scheme compared with HEVC benchmark.

Class	Sequence	BD-rate		
		*Y* (%)	*U* (%)	*V* (%)
Class B	Kimono	−1.1	0.1	0.2
	ParkScene	−0.4	−0.3	−0.3
	Cactus	−0.8	0.0	0.3
	BasketballDrive	−1.3	−0.2	−0.1
	BQTerrace	−3.2	−1.6	−1.6

Class C	BasketballDrill	−1.2	−0.6	0.2
	BQMall	−0.9	0.2	0.7
	PartyScene	0.2	0.5	0.3
	RaceHorses	−1.5	−0.5	−0.1

Class D	BasketballPass	−1.3	−0.4	0.3
	BQSquare	1.2	2.9	3.1
	BlowingBubbles	−0.3	0.4	0.8
	RaceHorses	−0.8	−0.9	0.0

Class E	FourPeople	−1.3	−0.4	0.1
	Johnny	−1.2	−0.4	−0.7
	KristenAndSara	−1.0	0.3	0.2

Class F	BasketballDrillText	−1.4	−0.2	0.1
	ChinaSpeed	−0.6	−0.5	−0.3
	SlideEditing	0.0	0.3	0.4
	SlideShow	−0.7	−0.1	−0.2

Class summary	Class B	−1.4	−0.4	−0.3
	Class C	−0.9	−0.1	0.3
	Class D	−0.3	0.5	1.0
	Class E	−1.2	−0.2	−0.1
	Class F	−0.7	−0.1	0.0

Overall	All	−0.9	−0.1	0.2

**Table 2:** BD-rate results compared with HEVC benchmark using image superresolution method.

Class	Sequence	BD-rate		
		*Y* (%)	*U* (%)	*V* (%)
Class C	BasketballDrill	0.8	1.2	2.1
	BQMall	2.8	2.7	3.0
	PartyScene	3.6	3.4	3.7
	RaceHorses	2.4	2.1	2.0

Class D	BasketballPass	1.7	1.3	2.0
	BQSquare	8.2	7.9	6.8
	BlowingBubbles	3.2	3.5	4.2
	RaceHorses	3.6	2.0	2.3

**Table 3:** BD-rate performance results using only the FRCNN-U model (LDB test conditions).

Class	*Y* (%)	*U* (%)	*V* (%)
Class B	−1.6	−0.4	−0.6
Class C	−1.9	−0.6	−0.6
Class D	−1.7	−1.0	−1.5
Class E	−3.7	0.2	−0.1
Class F	−1.7	−1.0	−0.7
Overall	−2.0	−0.6	−0.7

**Table 4:** Proportion (LDP) of the proposed FRCNN model in actual coding selection.

Class	QP 22 (%)	QP 27 (%)	QP 32 (%)	QP 37 (%)
Class B	67.13	50.52	32.93	26.28
Class C	55.60	47.75	31.72	27.05
Class D	54.26	41.92	23.40	20.17
Class E	54.53	37.88	18.18	12.43
Class F	25.76	21.81	26.57	21.20

**Table 5:** Comparison of running time.

Numbering	Data size/B	Number of targets	Traditional media art design		Bidirectional prediction FRCNN model	
			Running time/ms	Test results	Running time/ms	Test results
Image 1	512 × 300	8	13.245	Incorrect	11.525	Correct
Image 2	512 × 300	2	2.335	Correct	1.564	Correct
Image 3	512 × 300	6	8.956	Correct	7.213	Correct
Image 4	512 × 300	10	10.254	Correct	9.155	Correct
Image 5	512 × 300	2	3.625	Incorrect	1.145	Correct
Image 6	512 × 300	5	5.317	Correct	4.345	Correct
Image 7	512 × 300	6	11.175	Incorrect	10.354	Correct
Image 8	512 × 300	4	10.414	Correct	9.654	Correct
Image 9	512 × 300	6	7.256	Incorrect	6.215	Correct
Image 10	512 × 300	2	3.514	Correct	1.258	Correct

## Data Availability

The experimental data used to support the findings of this study are available from the corresponding author upon request.
